# Characteristics of nicotine product use, perceptions of dependence, and passive exposure among first-year university students in Brazil

**DOI:** 10.36416/1806-3756/e20240384

**Published:** 2025-05-21

**Authors:** Matheus Milani Silva, Sandra Silva Marques, Ludmila Candida de Braga, Suzana Erico Tanni, Ilda de Godoy, Irma de Godoy

**Affiliations:** 1. Faculdade de Medicina, Centro Universitário Claretiano, Rio Claro (SP) Brasil.; 2. Coordenação Estadual do Programa Nacional de Controle do Tabagismo, Secretaria de Saúde do Estado de São Paulo, São Paulo (SP) Brasil.; 3. Coordenadoria de Saúde, Universidade Estadual Paulista, São Paulo (SP) Brasil.; 4. Disciplina de Pneumologia, Departamento de Clínica Médica, Faculdade de Medicina de Botucatu, Universidade Estadual Paulista, Botucatu (SP) Brasil.; 5. Faculdade de Medicina, Centro Universitário Claretiano, Rio Claro (SP) Brasil.; 6. Disciplina de Pneumologia, Departamento de Clínica Médica, Faculdade de Medicina de Botucatu, Universidade Estadual Paulista, Botucatu (SP) Brasil.

**Keywords:** Cigarette smoking, Electronic nicotine delivery systems, Tobacco use disorder, Tobacco smoke pollution

## Abstract

**Objective:**

To assess nicotine product use among incoming students at public and private universities in Brazil.

**Methods:**

This was a multicenter cross-sectional study including a convenience sample of incoming university students (≥ 18 years of age). Current use of conventional cigarettes, electronic cigarettes, and other nicotine products, as well as indicators of dependence and knowledge of the consequences of active use of or passive exposure to nicotine products were investigated with the use of a 38-item questionnaire. Descriptive statistics were used to analyze the data.

**Results:**

A total of 863 students completed the questionnaire. Of those, most (61%) were female, and most (76.6%) identified themselves as White. In addition, most (86%) were in the 18- to 24-year age bracket, and most (88.6%) attended a public university. In the past 30 days, 20.7% reported using electronic cigarettes, 23.2% reported using conventional cigarettes, and more than 50% reported using other nicotine products. More than 80% agreed that electronic cigarettes are harmful to health and are as damaging as other types of smoking. Over half of the respondents reported using the product shortly after waking up, and, paradoxically, 81% stated that they would quit whenever they wanted. Passive exposure was reported by 21.6%, occurring at home (in 33.6%), in other indoor environments (in 41.7%), and in open environments (in 87.5%).

**Conclusions:**

Experimentation and current use of nicotine products are high among incoming students at public and private universities in Brazil. Passive exposure to nicotine products is commonplace. Advanced communication tools are needed, particularly to emphasize the dangers of nicotine dependence. Being a legal drug, nicotine is often perceived as harmless, reinforcing the misconception that it can be used without consequences.

## INTRODUCTION

Smoking has been considered the world’s greatest public health issue for decades. In the 20th century, 100 million premature deaths occurred as a result of tobacco use.^1^ The WHO estimates that more than 8 million people die prematurely each year from tobacco-related diseases.^2^ In fact, approximately 50% of smokers who do not quit die from tobacco-related diseases.^3^ ^,^ ^4^

A 2024 WHO report showed that smoking has been declining worldwide, with Brazil standing out globally with a 35% reduction since 2010.^5^ However, progress in tobacco control is under threat of regression because the tobacco industry has introduced new, noncombustible products, particularly electronic nicotine delivery systems (ENDS). 

Nicotine is the component of tobacco smoke that leads to addiction, and the nicotine content of ENDS has increased in more recent models, being as high as 60 mg/ml. When used in salt form or as analogs, it accelerates and enhances drug delivery to the brain at levels comparable to conventional cigarettes.^6^ In 2022, over 2.5 million young people, including 14.1% of U.S. high school students, were electronic cigarette users.^7^ Data from the National Youth Tobacco Survey show that prevalence was 3.1% and rose to 9.4% in 2022.^8^ National Youth Tobacco Survey data from students in the 11- to 17-year age bracket across 17 European cities revealed that the prevalence of electronic cigarette users doubled in some countries between 2014 and 2018, with rates ranging from 7.6% to 18.5%.^9^

In Brazil, ENDS have been banned since 2009. However, the COVITEL study-a telephone survey including 1,800 individuals from the five regions of the country-showed a lifetime prevalence of electronic cigarette use at 7.3% (95% CI, 6.0-8.9), being higher in men than in women, particularly among those in the 18- to 24-year age bracket and those with higher levels of education.^10^ Martins et al. assessed 711 medical students from the five regions of Brazil, reporting electronic cigarette experimentation and current use rates of 13.2% and 2.3%, respectively. The risk of experimentation was higher among those with higher socioeconomic status and those who had smokers in their social group.^11^

Although the tobacco industry claims that electronic cigarettes are 95% safer than conventional cigarettes, this statement is unsupported.^12^ An umbrella review that included data from 17 systematic reviews indicated a potential risk of cardiovascular effects of electronic cigarette use in smokers and nonsmokers. Short-term use has been linked to failure of pulmonary defense mechanisms, impacts on lung function, and increased airway resistance in smokers and nonsmokers.^13^ Users of flavored electronic cigarettes containing sugars, mint, menthol, or fruity flavors show the highest levels of DNA damage.^12^ ^,^ ^14^ Additional concerns include potential negative effects on brain development in young people using ENDS.^12^

In addition to the problems related to smoking any type of cigarette, nonsmokers who inhale the smoke released from the lit end of a cigarette or exhaled by smokers, as well as aerosols from ENDS, are subject to the same risks as active smokers. Passive or secondhand smokers are often unaware that there is no safe level of passive exposure and that even brief exposures can cause immediate harm. Passive exposure in adults leads to various life-threatening diseases, such as lung cancer, stroke, chronic obstructive pulmonary disease, and ischemic heart disease.^15^ In children, exposure is associated with an increased risk of sudden infant death syndrome, acute respiratory infections, otitis media, more severe asthma, long-term developmental issues, and reduced lung function, among others.^16^

WHO data estimate that 1.3 million nonsmokers exposed to secondhand smoke die prematurely each year.^2^ In a study evaluating the global prevalence of passive exposure among adolescents in the 12- to 16-year age bracket in 142 countries, it was reported that 62.9% were exposed on one or more days of the week; the exposure occurred at home, in public places, or other locations.^17^ In a study conducted across 18 countries in Latin America and the Caribbean, a passive exposure prevalence of 60.9% was reported, with no difference between boys and girls.^18^ Home is the primary location where children are exposed to cigarette smoke. In Australia, larger households, rural living, lower socioeconomic status, and single-parent households were risk factors for children’s exposure.^19^

In children, several factors are associated with greater susceptibility to tobacco smoke exposure, including a higher respiratory rate, a larger lung area, and the ongoing development process. In addition, they are generally unable to control their environment and, therefore, cannot avoid exposure to tobacco. Their lower ability to eliminate the chemicals in smoke that cause cancer makes them more susceptible to the effects of secondhand exposure.^20^ Although data on passive exposure to electronic cigarette aerosols is scarce, studies have provided evidence of respiratory changes^21^ and heavy metals in breast milk and umbilical cord blood, as well as in the urine and hair of children living with electronic cigarette smokers.^22^

Considering the severity of the consequences of active or passive exposure to any form of tobacco-derived products or nicotine-containing products, as well as the need to prevent addiction, we sought to characterize the use of and passive exposure to those products in students entering university, thus developing awareness, prevention, and treatment measures. In the present study we present results regarding nicotine product use and perceptions of dependence and passive exposure among first-year university students in Brazil. 

## METHODS

### Participants and procedures

We investigated nicotine product use in first-year students at the *Universidade Estadual Paulista* (Unesp, São Paulo State University), a public multicampus university in Brazil, and the *Centro Universitário Claretiano* School of Medicine, a private college located in the city of Rio Claro, Brazil, during the 2023-2024 academic years. We used a 38-item electronic questionnaire based on the Global Youth Tobacco Survey questionnaire.^23^ The questionnaire used in the present study is available in the supplementary material. We also investigated indicators of dependence; how the nicotine products were purchased or obtained; and participant knowledge of the consequences of active use of or passive exposure to nicotine products. The present study was conducted in strict accordance with the Declaration of Helsinki and was approved by the Unesp School of Medicine at Botucatu Research Ethics Committee (Reference no. 56582322.7.0000.5504). 

Undergraduate students ≥ 18 years of age attending Unesp or the *Centro Universitário Claretiano* School of Medicine were sent the questionnaire via email by the Unesp Office of the Dean for Undergraduate Studies, in compliance with the Brazilian National General Personal Data Protection Law (Law no. 13.709/2018). All participating patients gave written informed consent before completing the questionnaire. All responses were considered in the analysis. 

For statistical analysis, the proportions of each outcome were calculated for the sample as a whole and for each outcome separately in the public and private sectors, in accordance with the characteristics of the sample. 

## RESULTS

Although 1,155 students gave written informed consent, only 863 (74.7%) completed the questionnaire. Of those, most (61%) were female, and most (76.6%) identified themselves as White. In addition, most (86%) were in the 18- to 24-year age bracket, and most (88.6%) attended a public university. Undergraduate students in the humanities accounted for 40.5% of the study sample, the remaining 59.5% being in the sciences (biological sciences, 32%; and exact sciences, 27.5%). Nearly half (47%) of the respondents reported having less than 1,000 Brazilian reals per month for personal expenses, and the majority (> 75%) of parents or legal guardians had completed at least high school. Almost all participants (99.8%) had heard of electronic cigarettes. For any product, users were defined as those who reported using it at least once in the past 30 days. Experimentation of other tobacco products was assessed by the following question: Have you ever tried tobacco-derived products or tobacco products other than cigarettes (such as hookahs and hand-rolled cigarettes)? The proportions and characteristics of electronic cigarette users are presented in Table 1. Although most of the study participants recognized that electronic cigarettes are harmful, the prevalence of electronic cigarette use was found to be high, particularly at social events, and nearly 30% reported using electronic cigarettes on 10 or more days each month. Electronic cigarettes are widely available, with 30% of users reporting difficulty in avoiding smoking. Additionally, the use of hookahs or hand-rolled cigarettes was found to be common. 

**Table 1:** Characteristics of electronic cigarette users, as well as dependence indicators, opinions on potential harm, and concurrent use of conventional cigarettes.^a^

Variable	All	Public school students	Private school students
Prevalence	179 (20.7)	161 (21.0)	18 (18.4)
Place of use Home Social events Other	30 (16.7) 104 (58.1) 45 (25.2)	27(16.8) 96(59.6) 38(23.6)	3 (16.7) 8 (44.4) 7 (38.9)
Use in the last 30 days, no. of days 1-2 3-9 > 10	81 (45.3) 45 (25.1) 53 (29.6)	75(46.6) 39 (24.2) 47(29.2)	6 (33.3) 6 (33.3) 6 (33.4)
Place of purchase Tobacco shops Websites Other	68 (38.0) 71 (39.7) 40 (22.3)	61(37.9) 55(34.2) 45 (27.9)	7 (38.9) 6 (33.3) 5 (27.8)
Difficulty staying a week without using Easy/very easy Difficult/very difficult	146 (81.6) 33 (18.4)	131(81.4) 30(18.6)	15 (83.3) 3 (16.7)
Difficulty quitting Easy/very easy Difficult/very difficult	122 (68.2) 57 (31.8)	107(66.5) 54(33.5)	13 (72.2) 5 (27.8)
Experimentation with other forms of tobacco*	157 (87.7)	142 (88.2)	83.3 (85.7)
Use of other forms of tobacco in the last 30 days	95 (53.1)	90 (55.9)	6 (33.3)
ENDS are harmful to health Maybe yes/Maybe no/No Yes	25(14.0) 154 (86.0)	20(12.3) 141(87.0)	5 (27.8) 13 (72.2)
ENDS are as harmful as other forms of nicotine use Yes No	155 (86.5) 24 (13.4)	141(87.5) 20(12.5)	14 (77.8) 4 (22.2)

ENDS: electronic nicotine delivery systems. ^a^Data expressed as n (%). *Experimentation of other tobacco products was assessed by the following question: Have you ever tried tobacco-derived products or tobacco products other than cigarettes (such as hookahs and hand-rolled cigarettes)?

Regarding the use of conventional cigarettes, most (51.1%) reported that they had already tried them. The characteristics of conventional cigarette users and their use of other nicotine products, as well as dependence indicators and opinions on the potential harm caused by nicotine product use, are presented in Table 2. Conventional cigarettes remain very popular, their prevalence being higher than that of electronic cigarettes; more than half of users reported smoking conventional cigarettes on more than 10 days each month. The use of other tobacco products is also highly prevalent, and the majority reported that they consider electronic cigarettes harmful to health. 

**Table 2:** Characteristics of conventional cigarette users and their use of other nicotine products, as well as dependence indicators, opinions on potential harm, and concurrent use of electronic nicotine delivery systems.^a^

Variable	All	Public school students	Private school students
Prevalence	200 (23.2)	179 (23.4)	21 (21.4)
			
Use in the last 30 days, no. of days 1-2 3-9 >10	49 (24.5) 45 (22.5) 94 (53.0)	43 (24.0) 41 (22.9) 95 (53.1)	6 (28.6) 4 (19.0) 11 (52.4)
Age at initiation, years > 11 11-14 15-18 > 18	4 (2.0) 40 (20.0) 121 (60.5) 35 (17.5)	3 (16.7) 39 (21.8) 107 (59.8) 30 (16.8)	1 (4.8) 1 (4.8) 16 (76.2) 3 (14.2)
Experimentation with other forms of tobacco*	179 (89.5)	161 (89.9)	18 (85.7)
Use of other forms of tobacco in the last 30 days	111 (55.5)	105 (58.7)	6 (28.6)
ENDS are as harmful as other forms of nicotine use Yes No	169 (84.5) 31 (15.5)	155 (86.6) 24 (13.4)	15 (71.4) 6 (28.6)

ENDS: electronic nicotine delivery systems. ^a^Data expressed as n (%). *Experimentation of other tobacco products was assessed by the following question: Have you ever tried tobacco-derived products or tobacco products other than cigarettes (such as hookahs and hand-rolled cigarettes)?

**Figure 1:**
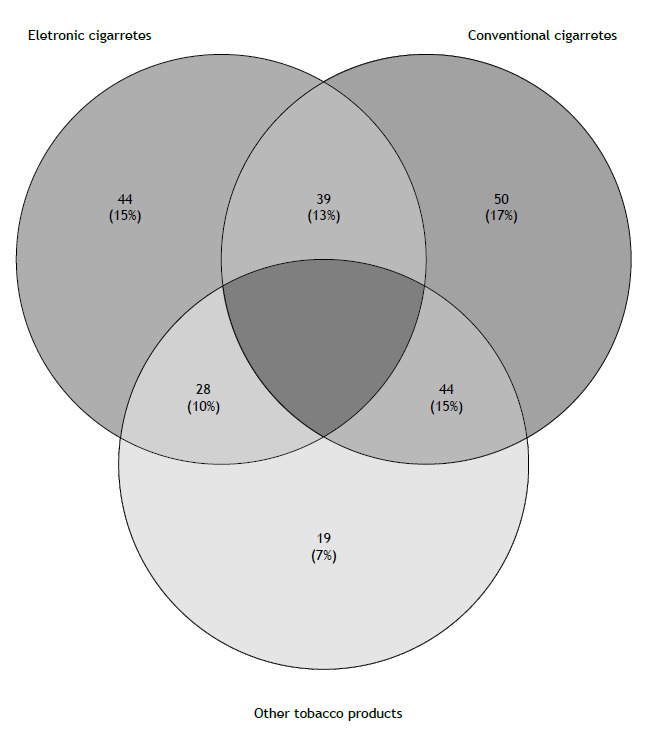
Venn diagram showing the numbers and proportions of users of electronic cigarettes, conventional cigarettes, and other tobacco products among first-year students at the São Paulo State University and the *Centro Universitário Claretiano* School of Medicine, both of which are located in the state of São Paulo, Brazil.

The combined use of ENDS and conventional cigarettes was reported by 53.5% of the conventional cigarette users and 59% of the ENDS users. Concurrent use of conventional cigarettes, electronic cigarettes, and other tobacco products (such as hookahs and roll-your-own cigarettes) in the last 30 days is shown in Figure 1. In this group of participants, the use of different tobacco products and combinations of different forms of smoking was very common. 

Of the total of ENDS users in the present study, 36.3% reported that they always or sometimes smoke shortly after waking up, as did 53.5% of conventional cigarette smokers. Nearly half of ENDS users (47.2%) and conventional cigarette smokers (48.2%) expressed an interest in quitting. Of the total of conventional cigarette smokers, 58% had been advised to quit, 50.5% had attempted to quit in the last 12 months, and 81% believed that they could quit if they wanted to. Of the total of ENDS users, 52.5% had been advised to quit, 46.3% had attempted to quit in the last 12 months, and 84.4% believed that they could quit if they wanted to. 

Of the 863 respondents, 21.6% reported to be unsure whether passive exposure to cigarette smoke or electronic cigarette aerosols causes harm, 33.6% reported passive exposure at home, 41.7% reported passive exposure in other indoor environments, and 85.7% reported passive exposure in open environments. Additionally, 89.9% had seen people smoking on the school/campus premises. Nearly half (42.5%) of the respondents reported that they support a ban on the use of nicotine products in open public spaces. 

## DISCUSSION

The main findings of the present study are a high proportion of nicotine product users among students entering university in Brazil; a high proportion of users of more than one product; a high rate of passive exposure in indoor environments; and a lack of awareness of nicotine dependence. Although a significant number of students show signs of dependence, the vast majority believe that they can quit tobacco whenever they choose. 

The proportions of nicotine product users reporting conventional cigarette or ENDS use at least once in the past 30 days were much higher in our study than in other studies conducted in Brazil,^10^ ^,^ ^11^ ^,^ ^24^ being 20.7% for ENDS and 22.3% for conventional cigarettes, with 30% of ENDS users and 53% of conventional cigarette users using them on more than 10 days in the past month. Additionally, over 50% of the public school students and approximately 30% of the private school students using ENDS or conventional cigarettes reported using other forms of tobacco in the past 30 days. In a study evaluating 711 medical students across the five regions of Brazil, the rates of current use were found to be 7.9% for conventional cigarettes, 11.4% for hookahs, and 2.3% for electronic cigarettes.^11^ In the COVITEL study,^10^ which evaluated 9,004 Brazilians ≥ 18 years of age, with 84.3% being > 24 years of age, the prevalence of conventional cigarette smoking was found to be 12.2%. The prevalence of daily or occasional ENDS use was 3.2% among men and 1.9% among women, whereas, for hookah use, it was 3.5% among men and 1.2% among women.^10^ Data from the 2019 Brazilian National Health Survey show that most electronic cigarette users (70%) are in the 15- to 24-year age bracket and approximately 90% are nonsmokers of conventional cigarettes.^24^ More than half of the nicotine product users in our study reported a combined use of conventional cigarettes and electronic cigarettes, with a high use in both groups. In the past 30 days, use of other nicotine products was above 50%, which is well above the rate reported in the COVITEL study.^10^

Data from the latest edition of the *Vigilância de Fatores de Risco e Proteção para Doenças Crônicas por Inquérito Telefônico* (VIGITEL, Telephone-based System for the Surveillance of Risk and Protective Factors for Chronic Diseases) show that, in individuals in the 18- to 24-year age bracket, the prevalence of active smoking is 6.7%, with 6.1% using electronic cigarettes.^25^ Analysis of data from the 2015 and 2019 Brazilian National School-Based Adolescent Health Surveys, which assessed students in the 13- to 17-year age bracket, shows that the proportion of students in Brazil who smoked in the 30 days prior to the survey was 6.8%, and the rate of experimentation with electronic cigarettes was 16.8%. The highest prevalence of (daily or occasional) electronic cigarette use was found among adult males, young adults in the 18- to 24-year age bracket, and individuals who had had ≥ 12 years of schooling.^26^

Possible explanations for the differences in the proportions of users of different nicotine products between the aforementioned studies and our study include the timing of data collection (between 2016 and early 2022), the characteristics of the sample, and the age groups included. Moreover, students usually undergo a period of significant stress before entering university and are therefore more susceptible to risk behaviors. 

In the present study, a substantial proportion of students reported passive exposure to nicotine products at home (33.6%), in other enclosed spaces (41.7%), and in open spaces (87.5%), with the vast majority (89.9%) witnessing people smoking on the school/campus premises. Additionally, a significant number are uncertain whether passive exposure causes health damage. Data from the 2023 VIGITEL show passive smoking at home at 9.9% and in the workplace at 8.9% among individuals in the 18- to 24-year age bracket.^25^ Among adolescents in the 12- to 16-year age bracket, the global prevalence in 142 countries was 62%, being 60.9% in Latin America.^18^ ^,^ ^27^ Therefore, the issue of passive exposure remains serious and widespread, and spreading awareness of the risks of passive smoking should be the subject of ongoing national campaigns. Although Brazilian Federal Law no. 12,546, issued on December 14, 2011, prohibits the use of cigarettes, cigars, cigarillos, pipes, or any other smoking product, whether tobacco-derived or not, in closed collective spaces, private or public, tobacco smoking of any kind and, consequently, exposure to smoke in private and public spaces is still prevalent and represents a significant public health problem. 

The perception of nicotine dependence is an interesting and concerning finding of the present study. The study population is familiar with electronic cigarettes (99.8%) and has tried them (51.5%), as well as knowing that they are harmful to health and as damaging as other forms of nicotine use (> 70%), with most parents having at least a high school education (> 75%). Signs of dependence (evidenced by smoking soon after waking) were present in 53.5%, yet interest in quitting tobacco is low (approximately 47%). The vast majority (84.4%) believe that they can quit whenever they want, indicating a misconception about the severity of nicotine dependence. Therefore, addressing aspects related to dependence is very important in this population. 

Our study has some limitations. We used the electronic platform of the Unesp School of Medicine at Botucatu, and the questionnaire was sent by email through the Unesp Office of the Dean for Undergraduate Studies. The email was sent three times at intervals of 2-3 weeks in 2023 and 2024. However, students rarely check their emails and are frequently invited to participate in studies, which may have affected adherence. Another possibility is that the respondents were primarily nonsmokers; nonetheless, the proportion of users was still very high. Additionally, Unesp has campuses in 24 cities, all of which are located in the state of São Paulo, and the *Centro Universitário Claretiano* School of Medicine is also located in the state of São Paulo; therefore, caution is required in generalizing the results. 

We conclude that rates of experimentation with and current use of electronic cigarettes and other nicotine products are high among incoming students at public and private universities in Brazil. Additionally, passive exposure to nicotine products in various settings is commonplace. Although knowledge gaps remain, awareness of the harms associated with nicotine dependence is widespread. However, merely knowing the risks does not prevent active or passive exposure. The use of nicotine products raises societal concerns, ongoing and tailored awareness initiatives being required for different social segments. More advanced communication tools are urgently needed, particularly to emphasize the dangers of nicotine dependence. Being a legal drug, nicotine is often perceived as harmless, reinforcing the misconception that it can be used without consequences. In response to these findings, the Unesp for a Nicotine-Free Generation program is being implemented across all university campuses, with full participation of students and professors, as well as official support from the university health office. 

## References

[1] Jha P (2009). Avoidable global cancer deaths and total deaths from smoking. Nat Rev Cancer.

[2] World Health Organization (WHO) [homepage on the Internet] (c2024). Tobacco.

[3] Banks E, Joshy G, Weber MF, Liu B, Grenfell R, Egger S (2015). Tobacco smoking and all-cause mortality in a large Australian cohort study findings from a mature epidemic with current low smoking prevalence. BMC Med.

[4] Siddiqi K, Husain S, Vidyasagaran A, Readshaw A, Mishu MP, Sheikh A (2020). Global burden of disease due to smokeless tobacco consumption in adults an updated analysis of data from 127 countries. BMC Med.

[5] World Health Organization (WHO) (2024). WHO global report on trends in prevalence of tobacco use 2000-2030.

[6] Wang X, Ghimire R, Shrestha SS, Borowiecki M, Emery S, Trivers KF (2023). Trends in Nicotine Strength in Electronic Cigarettes Sold in the United States by Flavor, Product Type, and Manufacturer, 2017-2022. Nicotine Tob Res.

[7] Cooper M, Park-Lee E, Ren C, Cornelius M, Jamal A, Cullen KA (2022). Notes from the Field E-cigarette Use Among Middle and High School Students - United States, 2022. MMWR Morb Mortal Wkly Rep.

[8] Mattingly DT, Hart JL (2024). Trends in Current Electronic Cigarette Use Among Youths by Age, Sex, and Race and Ethnicity. JAMA Netw Open.

[9] Tarasenko Y, Ciobanu A, Fayokun R, Lebedeva E, Commar A, Mauer-Stender K (2022). Electronic cigarette use among adolescents in 17 European study sites findings from the Global Youth Tobacco Survey. Eur J Public Health.

[10] Menezes AMB, Wehrmeister FC, Sardinha LMV, Paula PDCB, Costa TA, Crespo PA (2023). Use of electronic cigarettes and hookah in Brazil a new and emerging landscape. The COVITEL study, 2022. J Bras Pneumol.

[11] Martins SR, Araújo AJ, Wehrmeister FC, Freitas BM, Basso RG, Santana ANC (2023). Experimentation, current use of water pipe and electronic cigarette their associated factors among medical students a multicentric study in Brazil. J Bras Pneumol.

[12] Lang AE, Kathuria H, Braillon A, Ewart G, Dagli E, Stepp EL (2023). England Is Handing out E-Cigarettes Is the "Swap to Stop" Tobacco Control Scheme Harm Reduction or Harm Production?. Am J Respir Crit Care Med.

[13] Travis N, Knoll M, Cadham CJ, Cook S, Warner KE, Fleischer NL (2022). Health Effects of Electronic Cigarettes: An Umbrella Review and Methodological Considerations. Int J Environ Res Public Health.

[14] Braillon A, Lang AE (2023). The International Agency for Research on Cancer and e-cigarette carcinogenicity time for an evaluation. Eur J Epidemiol.

[15] Drope J, Hamill S, Chaloupka F, Guerrero C, Lee H, Mirza M (2022). The Tobacco Atlas.

[16] Campbell M, Winnall W, Ford C, Winstanley M (2021). Tobacco in Australia: Facts and issues [Internet].

[17] Ma C, Yang H, Zhao M, Magnussen CG, Xi B (2022). Prevalence of waterpipe smoking and its associated factors among adolescents aged 12-16 years in 73 countries/territories. Front Public Health.

[18] Bernabe-Ortiz A, Carrillo-Larco RM (2023). Second-hand smoke exposure in adolescents in Latin America and the Caribbean a pooled analysis. Lancet Reg Health Am.

[19] Longman JM, Passey ME (2013). Children, smoking households and exposure to second-hand smoke in the home in rural Australia: analysis of a national cross-sectional survey. BMJ Open.

[20] Chao MR, Cooke MS, Kuo CY, Pan CH, Liu HH, Yang HJ (2018). Children are particularly vulnerable to environmental tobacco smoke exposure Evidence from biomarkers of tobacco-specific nitrosamines, and oxidative stress. Environ Int.

[21] Islam T, Braymiller J, Eckel SP, Liu F, Tackett AP, Rebuli ME (2022). Secondhand nicotine vaping at home and respiratory symptoms in young adults. Thorax.

[22] Ballbè M, Fu M, Masana G, Pérez-Ortuño R, Gual A, Gil F (2023). Passive exposure to electronic cigarette aerosol in pregnancy A case study of a family. Environ Res.

[23] Centers for Disease Control and Prevention (2014). Global Youth Tobacco Survey Collaborative Group. Global Youth Tobacco Survey (GYTS): Core Questionnaire with Optional Questions, Version 1.2.

[24] Bertoni N, Cavalcante TM, Souza MC, Szklo AS (2021). Prevalence of electronic nicotine delivery systems and waterpipe use in Brazil where are we going?. Rev Bras Epidemiol.

[25] Brasil. Ministério da Saúde. Secretaria de Vigilância em Saúde e Ambiente, Departamento de Análise Epidemiológica e Vigilância de Doenças Não Transmissíveis [homepage on the Internet] Vigitel Brasil 2023: Vigilância de Fatores de Risco e Proteção para Doenças Crônicas por Inquérito Telefônico: Estimativas sobre Frequência e Distribuição Sociodemográfica de Fatores de Risco e Proteção para Doenças Crônicas nas Capitais dos 26 Estados Brasileiros e no Distrito federal em 2023.

[26] Malta DC, Gomes CS, Alves FTA, Oliveira PPV, Freitas PC, Andreazzi M (2022). The use of cigarettes, hookahs, electronic cigarettes, and other tobacco indicators among Brazilian schoolchildren data from National School Health Survey 2019. Rev Bras Epidemiol.

[27] Ma C, Heiland EG, Li Z, Zhao M, Liang Y, Xi B (2021). Global trends in the prevalence of secondhand smoke exposure among adolescents aged 12-16 years from 1999 to 2018: an analysis of repeated cross-sectional surveys. Lancet Glob Health.

